# Aortic Root Dilatation in Mucopolysaccharidosis I–VII

**DOI:** 10.3390/ijms17122004

**Published:** 2016-11-29

**Authors:** Meena Bolourchi, Pierangelo Renella, Raymond Y. Wang

**Affiliations:** 1 Department of Pediatrics, Children’s Hospital of Orange County, Orange, CA 92868, USA; meena.bolourchi@gmail.com (M.B.); prenella@choc.org (P.R.); 2 Department of Pediatrics, University of California-Irvine School of Medicine, Orange, CA 92868, USA; 3 Pediatric Heart Institute, Children’s Hospital of Orange County, Orange, CA 92868, USA; 4 Division of Metabolic Disorders, Children’s Hospital of Orange County, Orange, CA 92868, USA

**Keywords:** aortic root dilatation, mucopolysaccharidosis, echocardiogram, enzyme replacement therapy, z-scores

## Abstract

The prevalence of aortic root dilatation (ARD) in mucopolysaccharidosis (MPS) is not well documented. We investigated aortic root measurements in 34 MPS patients at the Children’s Hospital of Orange County (CHOC). The diagnosis, treatment status, age, gender, height, weight and aortic root parameters (aortic valve annulus (AVA), sinuses of Valsalva (SoV), and sinotubular junction (STJ)) were extracted by retrospective chart review and echocardiographic measurements. Descriptive statistics, ANOVA, and paired post-hoc *t*-tests were used to summarize the aortic dimensions. Exact binomial 95% confidence intervals (CIs) were constructed for ARD, defined as a *z-*score greater than 2 at the SoV. The patient age ranged from 3.4–25.9 years (mean 13.3 ± 6.1), the height from 0.87–1.62 meters (mean 1.24 ± 0.21), and the weight from 14.1–84.5 kg (mean 34.4 ± 18.0). The prevalence of dilation at the AVA was 41% (14/34; 95% CI: 25%–59%); at the SoV was 35% (12/34; 95% CI: 20%–54%); and at the STJ was 30% (9/30; 95% CI: 15%–49%). The highest prevalence of ARD was in MPS IVa (87.5%). There was no significant difference between mean z-scores of MPS patients who received treatment with hematopoietic stem cell transplantation (HSCT) or enzyme replacement therapy (ERT) vs. untreated MPS patients at the AVA (*z* = 1.9 ± 2.5 vs. *z* = 1.5 ± 2.4; *p* = 0.62), SoV (*z* = 1.2 ± 1.6 vs. *z* = 1.3 ± 2.2; *p* = 0.79), or STJ (*z* = 1.0 ± 1.8 vs. *z* = 1.2 ± 1.6; *p* = 0.83). The prevalence of ARD was 35% in our cohort of MPS I–VII patients. Thus, we recommend screening for ARD on a routine basis in this patient population.

## 1. Introduction

Aortic root dilatation (ARD) typically develops via cystic medial degeneration and migration of smooth muscle cells from the tunica media to the tunica intima with age [1]. In children with connective tissue diseases, such as Marfan or Ehlers-Danlos syndromes, this cystic medial necrosis occurs at a much younger age due to defective fibrillin-1 or collagen production, respectively. Aortic root dilatation was found in up to 60% of adult patients with Marfan syndrome, and was due to fragmentation of the elastin in the tunica media [2]. ARD can lead to aortic aneurysm formation and aortic dissection, contributing to significant morbidity and mortality [3,4]. Patients with mucopolysaccharidosis (MPS) types I–VII develop multi-organ dysfunction, including progressive cardiovascular diseases such as valve stenosis and/or regurgitation, left ventricular hypertrophy, and congestive heart failure due to the absence of enzymes needed for the degradation of glycosaminoglycans (GAGs) [5]. These GAGs, which are polar glycan polymers predominantly found in extracellular matrix, deposit within valvular interstitial cells in histopathological studies of human and murine MPS I models [6]. In dog models of MPS, ARD has been associated with the deposition of GAGs in the medial layer of the aorta with subsequent infiltration of activated macrophages, with fragmentation and loss of elastin laminae [7]. In feline models of MPS I and VI, the aortic diameter was significantly enlarged as compared to unaffected controls [8]. In human patients with severe untreated MPS I less than one year of age, aortic root dilatation was present in five of 13 (38%) untreated patients [9]. While treatment in the form of hematopoietic stem cell transplantation (HSCT) or enzyme replacement therapy (ERT) exists for all types of MPS except types III, IVb, and VII, the prevalence of ARD in MPS patients in the post-treatment era is not well documented. Herein, we report aortic root dimensions from a single-center cohort of 34 MPS patients.

## 2. Results

Echocardiograms of 34 patients (38% female, age 13.3 ± 6.1 years, range 3.4 to 25.9 years) with MPS I-VII were reviewed in our study. Patient heights ranged from 0.87–1.62 m (mean 1.24 ± 0.21 m) and weights ranged from 14.1–84.5 kg (mean 34.4 ± 18.0 kg). Patients with MPS I, II, IVa, except one, and VI (*n* = 21) received ERT and/or HSCT. Patients with MPS IIIa, IIIb, IVb, VII (due to a lack of approved therapies) and one patient with IVa (patient declined ERT) were untreated (*n* = 13; see Table 1).

**Figure 1:**
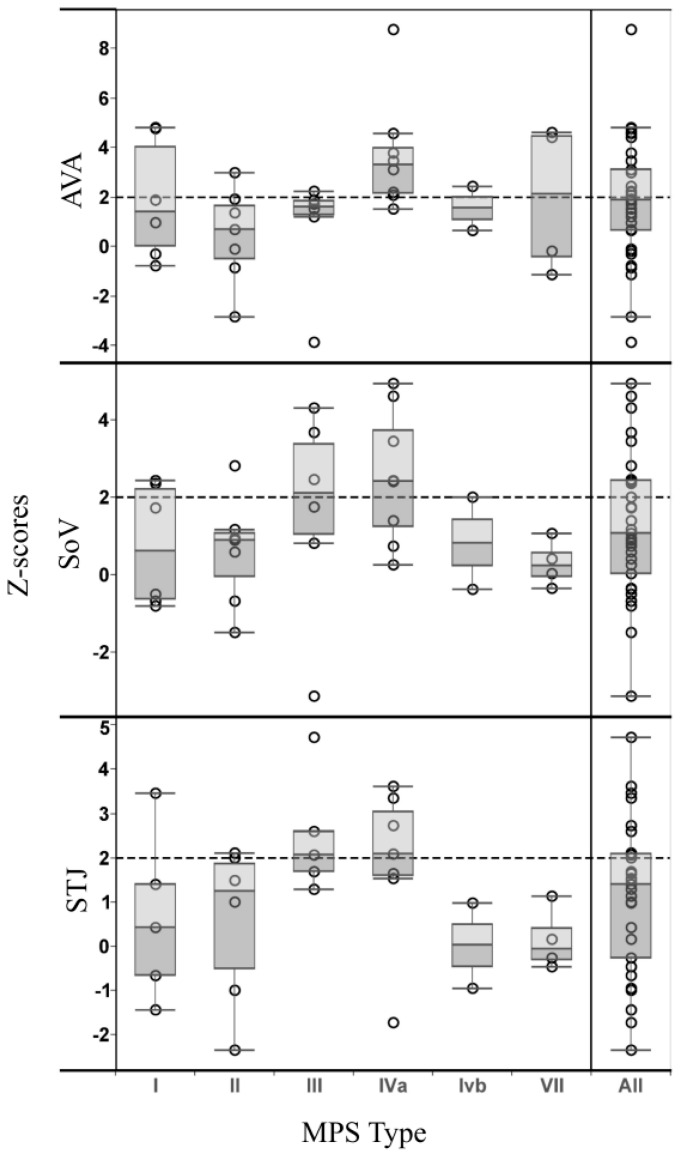
Z-scores of aortic root measurements for subtypes of mucopolysaccharidosis patients in a Tukey boxplot. The open circles represent the z-score of each individual patient at the AVA (aortic valve annulus), SoV (sinuses of Valsalva), and STJ (sinotubular junction). The 25–75th percent interquartile range is represented by the edges of the box. The circular points above the dotted line indicate aortic root measurements that exceed the 97th percentile of normal measurements.

There was no significant difference in age (*p* = 0.45), weight (*p* = 0.14), BSA (*p* = 0.09), AVA (*p* = 0.21), SoV (*p* = 0.29), or STJ (*p* = 0.21) between MPS groups I–VII; however, height significantly differed between MPS types (*p* = 0.05). MPS IVa patients were shorter than MPS II patients (*p* = 0.04), MPS III patients (*p* = 0.02), and MPS VII patients (*p* = 0.01). Furthermore, there was a baseline difference in age (*p* = 0.04) and height (*p* = 0.01) for treated vs. untreated MPS patients, but no difference in weight, BSA, AVA, SoV, or STJ. Dilation at the AVA was found in 14 of 34 patients (41%, 95% CI: 25%–59%); dilation at the level of the SoV occurred in 12 of 34 patients (35%, 95% CI: 20%–54%); and STJ enlargement was present in nine of 30 patients (30%, 95% CI: 15%–49%) as depicted in Figure 1. The STJ measurement was not included in four of 34 patients due to poor image quality. ARD was observed in all MPS types except for type VI, where there was only one patient in that subgroup. The highest prevalence of ARD was in MPS IVa, where five of eight patients (62.5%) had z-scores > 2 at the SoV.

Using ANOVA to compare the z-scores for the aortic root measurements for MPS I–VII, there was no statistical difference between the MPS types at the AVA (*p* = 0.21), the SoV (*p* = 0.29) or the STJ (*p* = 0.92). Subgroup analysis comparing MPS types using post-hoc t-tests indicated the z-scores were significantly larger at the AVA for MPS IVa vs. II (3.7 ± 2.3 vs. 0.4 ± 1.9, respectively; *p* = 0.01) and for IVa vs. III (3.7 ± 2.3 vs. 0.8 ± 2.3, respectively; *p* = 0.04), and larger at the SoV between MPS IVa vs. II (2.5 ± 1.7 vs. 0.6 ± 1.4, respectively; *p* = 0.03) and IVa vs. VII (2.5 ± 1.7 vs. 0.3 ± 0.6, respectively; *p* = 0.03). There was no significant difference in z-scores between any of the MPS types at the STJ. There was also no significant difference between mean z-scores of the treated vs. untreated patients at the AVA (treated *z* = 1.9 ± 2.5 vs. untreated *z* = 1.5 ± 2.4; *p* = 0.62), SoV (treated *z* = 1.2 ± 1.6 vs. untreated *z* = 1.3 ± 2.2; *p* = 0.79), or STJ (treated *z* = 1.0 ± 1.8 vs. untreated *z* = 1.2 ± 1.6; *p* = 0.83).

## 3. Discussion

This relatively large single-center retrospective study of 34 patients with mucopolysaccharidosis I–VII showed that the overall prevalence of ARD was 35.3% (12 of 34 patients had a z-score > 2 at the SoV). In addition, many patients also had abnormally enlarged aortic annuli and sinotubular junction diameters. It is unclear why some patients only had dilatation at one or two of the three aortic root parameters. Perhaps thickening of the valve in some patients may cause progressive aortic regurgitation, thus stretching the aortic valve annulus, leading to a feed-forward phenomenon that further worsens valve dysfunction and ARD. Prior reports and one human case study of severe MPS I have demonstrated aortic root abnormalities [9,10,11]. In that study, 5 of 13 (38%) of severe, untreated MPS I patients had ARD [9]. While our study also identified a high prevalence of ARD in the MPS I patients (2 of 4 severe cases, and 2 of 6 overall), we also found that the MPS subtype with the highest prevalence of ARD was MPS IVa (62.5%, 5 of 8 patients). The presence of coronary and aortic elastin fibril fragmentation in post-mortem histopathologic assessments of MPS I [12] and MPS IVa [11] patients, as well as MPS animal models, may suggest a link to the relatively common finding of ARD in our study.

Further prospective studies are needed to determine if ARD in MPS patients is progressive and/or leads to increased morbidity or mortality from vascular events such as aortic dissection. None of the patients in our cohort have had aortic dissection. This study did not show any difference in ARD between the treated group (ERT or HSCT) vs. the untreated group. While no specific studies have been conducted to assess the effect of treatment on aortic root measurements in MPS patients, this observation highlights the previously documented refractory nature of MPS cardiac manifestations in spite of treatment [13]. Some refractory factors are valvular thickening [9] or intimal medial thickness [14]. A suboptimal treatment effect with respect to the cardiovascular system is likely the result of limited penetration of the circulating enzyme into valve and vascular tissues, even at supra-physiologic levels [15].

Although we report on a relatively large cohort of MPS patients, the number of patients in each subtype of MPS was small. In addition, the post hoc nature of comparing such small populations is a limitation of this study. Furthermore, these results may have limited generalizability, as all the patients came from a single center. Another confounding factor was the time elapsed between the start of ERT or HSCT and echocardiographic assessments. Additionally, indexing measurements by BSA is designed for patients of normal stature, and the bias of z-scores due to the increasing body mass index in pediatric patients has been recognized [16]. As MPS patients, especially those with MPS IVa, tend to have a disproportionately short stature, their z-scores for aortic measurements, which are normalized to BSA, may have been overestimated. For example, Marfan syndrome patients are taller on average than their unaffected peers and thus separate z-score measurements based on height instead of BSA have been used in that patient population [17]. A vital area of future investigation is the development of aortic z-scores specifically for the MPS patient population.

## 4. Materials and Methods

**Figure 2:**
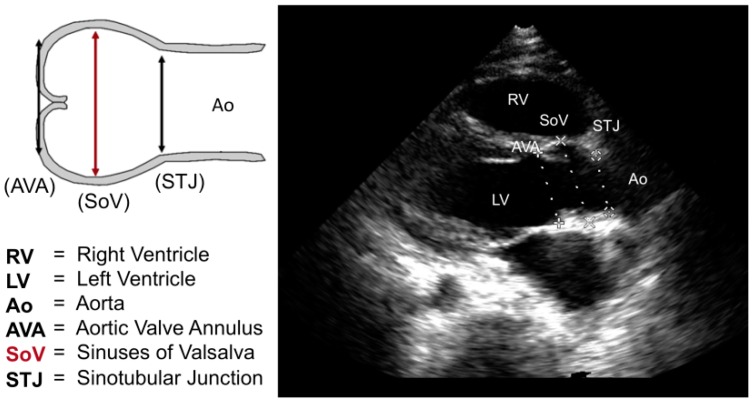
Z Aortic root measurements via schematic and echocardiography at the parasternal long-axis view.

We conducted an anonymized, retrospective chart review of 34 patients with enzymatically or molecularly confirmed mucopolysaccharidoses evaluated at Children’s Hospital of Orange County (CHOC). The study (IRB #1409104) was approved by the CHOC institutional review board (8 October 2014). As the study was retrospective and anonymized, the IRB approved our request for waiver of informed consent. The MPS type, treatment status, age, gender, height, and weight were extracted from the most recent cardiology clinic outpatient visit note during which an echocardiogram was performed. The body surface area (BSA) in square meters was calculated using the Haycock method [18]. The most recent echocardiogram for each patient was reviewed and measurements from the proximal aorta at the aortic valve annulus (AVA), sinuses of Valsalva (SoV), and sinotubular junction (STJ) were recorded from the parasternal long axis view according to published guidelines [19] (see Figure 2).

Echocardiographic studies were performed using iE33 cardiac ultrasound systems (Philips Medical Systems, Andover, MA, USA) according to the standard laboratory protocol. The S-8 and S-5 transducers were most often utilized and acquisition parameters were optimized according to patient size and acoustic windows. Harmonic imaging was often used to optimize signal-to-noise ratio and to enhance visualization of the relevant cardiac structures. Aortic root measurements were indexed utilizing the ratio of the absolute measurement to the patient’s BSA. Z-scores were derived according to Colan et al 2006 for AVA and SoV [20] and Daubeney et al 1999 for STJ [21]. ARD was defined as a z-score ≥ 2 at SoV. Results are reported as mean ± standard deviation for continuous variables and mean (95% confidence interval (CI)) for discrete variables.

To determine if there was a difference in aortic root measurements between each MPS type, we used ANOVA to compare the z-scores and set statistical significance at a two-sided *p*-value of 0.05. We then performed further subgroup analysis using paired post-hoc *t*-tests. Finally, we divided the patients into treated and untreated, and performed paired *t*-test analysis to determine if any aortic root parameters differed according to treatment status.

## 5. Conclusions

Aotic root dilatation is highly prevalent in mucopolysaccharidoses and routine screening for this potentially important finding should be incorporated into the multidisciplinary care of MPS patients.

## Figures and Tables

**Table 1:** Patient demographics: the age refers to the age of the patient at the time of his or her latest echocardiogram. BSA = body surface area, AVA = aortic valve annulus, SoV = sinuses of Valsalva, Std = standard deviation. STJ = sinotubular junction.

Demographics and Echocardiographic Parameters	MPS Type																						
	All Patients			I			II			III			IVa			IVb			VI		VII		
	*n*	Mean	Std	*n*	Mean	Std	*n*	Mean	Std	*n*	Mean	Std	*n*	Mean	Std	*n*	Mean	Std	*n*	Mean	*n*	Mean	Std
Age (years)	34	13.0	6.1	6	9.5	3.5	7	14.2	6.9	6	16.4	4.9	8	12.3	7.9	2	9.1	4.5	1	9.9	4	15.5	5.2
Height (cm)	34	123.9	21.4	6	115.9	28.2	7	131.2	16.6	6	136.2	14.9	8	110.2	18.8	2	119.5	19.1	1	95.0	4	142.0	8.2
Weight (kg)	34	34.4	18.0	6	28.8	14.7	7	41.7	23.7	6	36.8	17.3	8	26.0	13.7	2	24.6	7.9	1	17.1	4	52.8	11.7
BSA (m^2^)	34	1.1	0.4	6	1.0	0.3	7	1.2	0.4	6	1.2	0.3	8	0.9	0.3	2	0.9	0.2	1	0.7	4	1.5	0.2
AVA (cm)	34	1.8	0.4	6	1.7	0.2	7	1.7	0.3	6	1.8	0.4	8	1.9	0.5	2	1.7	0.4	1	1.5	4	2.2	0.5
SoV (cm)	34	2.3	0.5	6	2.1	0.2	7	2.3	0.4	6	2.5	0.6	8	2.4	0.7	2	2.1	0.6	1	1.7	4	2.5	0.3
STJ (cm)	32	1.9	0.5	5	1.5	0.7	7	1.9	0.4	5	2.3	0.3	8	2.0	0.5	2	1.7	0.4	1	1.4	4	2.1	0.3
z-score (AVA)	34	1.8	2.4	6	1.9	2.4	7	0.4	1.9	6	0.8	2.3	8	3.7	2.3	2	1.5	1.3	1	1.7	4	1.9	3.0
z-score (SoV)	34	1.2	1.8	6	0.8	1.6	7	0.6	1.4	6	1.6	2.7	8	2.5	1.7	2	0.8	1.7	1	0.0	4	0.3	0.6
z-score (STJ)	30	1.1	1.7	5	0.6	1.9	6	0.5	1.8	5	2.5	1.4	7	1.9	1.8	2	0.0	1.4	1	0.0	4	0.1	0.7

## Abbreviations

ARD	Aortic root dilatation
AVA	Aortic valve annulus
BSA	Body surface area
CHOC	Children’s Hospital of Orange County
CI	Confidence interval
ERT	Enzyme replacement therapy
GAGs	Glycosaminoglycans
HSCT	Hematopoietic stem cell transplantation
MPS	Mucopolysaccharidosis
SoV	Sinuses of valsalva
STJ	Sinotubular junction
